# Mesenchymal stromal cells cultured in physiological conditions sustain citrate secretion with glutamate anaplerosis

**DOI:** 10.1016/j.molmet.2022.101532

**Published:** 2022-06-22

**Authors:** Giuseppe Taurino, Ruhi Deshmukh, Victor H. Villar, Martina Chiu, Robin Shaw, Ann Hedley, Engy Shokry, David Sumpton, Erica Dander, Giovanna D'Amico, Ovidio Bussolati, Saverio Tardito

**Affiliations:** 1 Laboratory of General Pathology, Dept. of Medicine and Surgery, University of Parma, 43125, Parma, Italy; 2 Cancer Research UK Beatson Institute, Garscube Estate, Switchback Road, Glasgow, G61 1BD, UK; 3 Centro Ricerca Tettamanti, Pediatric Dept., University of Milano-Bicocca, Fondazione MBBM, Monza, 20900, Italy; 4 MRH - Microbiome Research Hub, Parco Area delle Scienze 11/A, University of Parma, 43124 Parma, Italy; 5 Institute of Cancer Sciences, University of Glasgow, Glasgow, G61 1QH, UK

**Keywords:** Mesenchymal stromal cells, Metabolism, Physiological medium, Plasmax, Hypoxia, Stable isotope tracing, Primary cells, Glutamine, Citrate, Glutamate

## Abstract

Bone marrow mesenchymal stromal cells (MSCs) have immunomodulatory and regenerative potential. However, culture conditions govern their metabolic processes and therapeutic efficacy. Here we show that culturing donor-derived MSCs in Plasmax™, a physiological medium with the concentrations of nutrients found in human plasma, supports their proliferation and stemness, and prevents the nutritional stress induced by the conventional medium DMEM. The quantification of the exchange rates of metabolites between cells and medium, untargeted metabolomics, stable isotope tracing and transcriptomic analysis, performed at physiologically relevant oxygen concentrations (1%O_2_), reveal that MSCs rely on a high rate of glucose to lactate conversion, coupled with parallel anaplerotic fluxes from glutamine and glutamate to support citrate synthesis and secretion. These distinctive traits of MSCs shape the metabolic microenvironment of the bone marrow niche and can influence nutrient cross-talks under physiological and pathological conditions.

## Highlights

• Physiological nutrient concentrations support MSCs proliferation and stemness *in vitro*.
• ATF4 levels are higher in primary MSCs cultured in DMEM than in Plasmax.
• MSCs are highly glycolytic and use glutamine and glutamate for anaplerosis.
• The high affinity glutamate uptake of MSCs supports citrate synthesis and secretion.

## Graphical abstract

**Figure:**
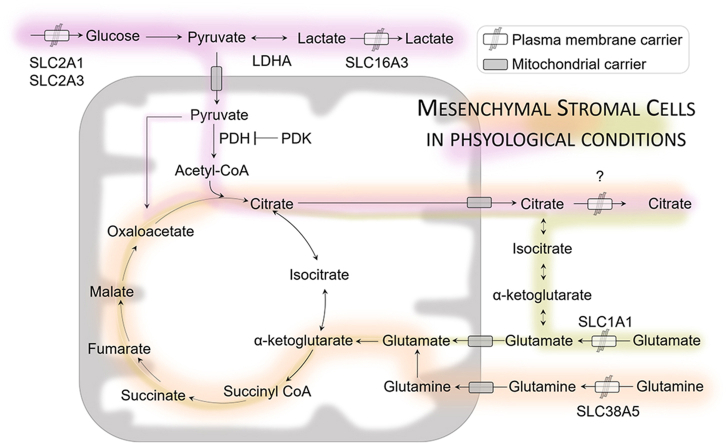

## 1 Introduction

Mesenchymal stromal cells (MSCs) are multipotent stem cells located in several tissues such as the bone marrow niche, the umbilical cord and adipose tissue. According to the International Society for Cellular Therapy, human MSCs should grow as adherent cultures, have the potential to differentiate into adipocytes, chondroblasts and osteoblasts, and express the surface markers CD105, CD73, CD90, while being negative for CD11b, HLA-DR, CD14, CD19, CD34, CD45, CD79α [1]. Depending on their tissue of origin, MSCs can also differentiate into myocytes, cardiomyocytes, hepatocytes and neurons [2].

MSCs lack class II MHC expression, have immunomodulatory paracrine functions mediated by the release of cytokines and exosomes, and promote wound healing and tissue regenerative processes [3]. For these reasons, MSCs are widely employed in cell therapy for allogeneic transplants in regenerative medicine [4], and to treat autoimmune, heart, musculoskeletal and nervous system diseases [5]. MSCs are also used in the treatment of neonatal disorders such as bronchopulmonary dysplasia, intraventricular hemorrhage and hypoxic-ischemic encephalopathy [6]. Recently, it has been proposed to reprogram the MSCs of the tumor microenvironment towards anticancer functions [7], and to exploit their immunomodulatory activity in patients with SARS-CoV2 infection [8]. For these therapeutic interventions, MSCs are generally sourced from adipose tissue and the bone marrow, and primary cultures are expanded *in vitro* to reach suitable numbers for clinical applications [3]. However, historic non-physiological culture conditions are known to impact cell metabolic and signaling states [[9], [10], [11]], thus potentially altering the functional properties of MSCs. For instance, culturing MSCs with low oxygen levels relevant to the bone marrow niche (1–5%) [12] dampens oxidative stress [13], while promoting proliferation [14], the expression of stemness markers [15], chondrogenic differentiation [16], immunomodulatory effects [17], and efficacy of MSC-based therapy for the treatment of ischemic diseases [18]. Furthermore, oxygen concentration directly impacts MSCs' metabolism, in particular the glycolytic flux [19], lactate secretion [13] and glutamine-dependent synthesis of tricarboxylic acids (TCA) cycle intermediates [20]. However, the metabolism of MSCs has never been studied at physiological nutrient concentrations.

Here we have quantified the exchange rates of nutrients and metabolites between media and MSCs, and profiled the metabolic fluxes of glucose and glutamine carbons in a panel of eight primary bone marrow-derived MSCs cultured at 1% oxygen in Plasmax™, a physiological culture medium with concentrations of nutrients resembling those found in human plasma.

## 2 Results

### 2.1 The metabolic profile of MSCs cultured with physiological levels of nutrients

**Figure 1:**
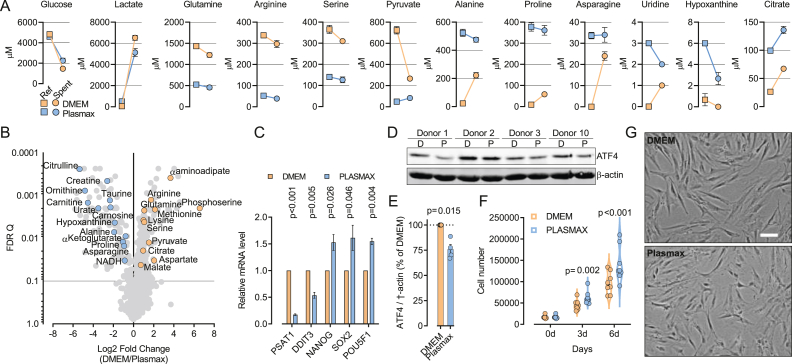
The metabolic profile of MSCs cultured with physiological levels of nutrients. (A–C) MSCs incubated in Plasmax or DMEM at 21% O_2_ for 48 h, and the levels of metabolites in the reference and spent media (A), in intracellular extracts (B), and gene expression (C) were measured. (A) The concentrations (μM) of metabolites were measured in reference medium (Ref) or in medium incubated with cells (Spent). During the 48 h incubation the average values of μg prot/well were not significantly different between Plasmax and DMEM. Data points are means ± SD from 3 wells derived from 1 donor. (B) Untargeted analysis of intracellular metabolites with significantly different levels in cells incubated in DMEM or Plasmax. Orange circles represent metabolites more abundant in DMEM-cultured cells. Blue circles represent metabolites more abundant in Plasmax-cultured cells. Data points are means from 3 wells derived from 1 donor. q values refer to a false discovery rate approach corrected for multiple comparisons with a two-stage step-up method of Benjamini, Krieger and Yekutieli. (C) The relative expression of nutritional stress- and stemness-markers evaluated by RT-PCR. Values were normalized to *RPL15* expression and are shown as ratios of the mean values obtained in DMEM-cultured cells. p-values were calculated with a one sample t-test with reference value = 1. Bars are means ± SD from 3 wells derived from 1 donor. (D) Western blot of ATF4 from extracts of MSCs cultured in DMEM or Plasmax at 21% O_2_ for 48 h. β Actin is shown as loading control. (E) Quantification of the Western Blot shown in (D). Data points are means of values obtained with MSCs lines derived from 4 donors. p value was calculated with a one sample t-test with reference value = 100. (F) Cell number of MSCs incubated in DMEM or Plasmax for 6 days. Data points are means of values obtained with MSCs lines derived from 8 donors. p values refer to a two-tailed Student's t-test for paired samples. (G) Representative images of MSCs cultured for 48 h in DMEM or Plasmax (bar = 100 μm).

Historic cell culture media, such as DMEM, lack several non-essential nutrients normally available in human plasma. Conversely, most of the nutrients present in DMEM largely exceed the concentrations found in human plasma. Providing cultured cells with nutrients at concentrations found in human plasma should allow a more relevant profiling of their consumption and secretion. To address this point, primary MSCs were cultured for 48 h in Plasmax or in low-glucose (5.56 mM) DMEM, and nutrient concentrations were quantified in reference and spent media (Figure 1A).

Glucose was the nutrient most avidly consumed by primary MSCs in both DMEM and Plasmax. Despite the comparable concentrations of glucose in the two media, MSCs consumed more glucose when cultured in DMEM, where they released more lactate. The supra-physiological concentration of glutamine, arginine, serine and pyruvate found in DMEM boosted their consumptions compared to Plasmax. Conversely, non-essential amino acids absent from the DMEM formulation, such as alanine, proline and asparagine, were secreted by MSCs cultured in DMEM but not in Plasmax. Uridine and hypoxanthine can be salvaged for the biosynthesis of nucleotides, and they were avidly consumed from Plasmax despite their low initial concentrations (3 μM and 6 μM, respectively). Unexpectedly, MSCs secreted citrate into the medium, a behavior previously reported only for astrocytes and specialized cells of the prostate epithelium [21]. Notably, the outward flux of the tricarboxylic acid was observed even in Plasmax, where its initial concentration is 100 μM, suggesting an energized mechanism of efflux.

To quantify the effects of the different culture media on intracellular metabolism, an untargeted metabolomics analysis was performed comparing MSCs cultured in DMEM with those cultured in Plasmax. Most of the components selectively present in Plasmax were enriched in cells cultured in this medium compared to DMEM (Figure 1B). Amongst the metabolites enriched in DMEM-cultured MSCs, phosphoserine was the most upregulated. Phosphoserine is an intermediate of serine biosynthesis, a pathway activated by serine restriction in several cancer cell types [22]. However, serine was >3 fold higher in DMEM than in Plasmax (Figure 1A), resulting in significantly higher intracellular levels (Figure 1B). Therefore, we hypothesized that the paradoxical activation of the serine biosynthetic pathway observed in MSCs cultured in DMEM could be caused by the lack of several non-essential amino acids (proline, asparagine, alanine) in this medium (Figure 1A) that lowered their intracellular levels (Figure 1B). In fact, the phosphoserine producing enzyme, PSAT1, is a transcriptional target of the GCN2-eIF2alpha-ATF4 signaling axis, which is activated in response to amino acid deprivation [23]. Consistently with this hypothesis, the expression of ATF4 protein and its targets *PSAT1* and *DDIT3* (CHOP) [24], were increased in MSCs cultured in DMEM compared to Plasmax (Figure 1C–E). Moreover, we found that the expression of the stem cell markers *NANOG*, *SOX2*, and *POUF5F1* was increased in MSCs when cultured in Plasmax compared to DMEM (Figure 1C), consolidating the notion that a physiological metabolic state preserves stemness in MSCs. To test if the different nutrient availability in the two media could affect proliferation, we cultured MSCs derived from eight donors in DMEM or Plasmax (Figure 1F). While the morphology of the cells was comparable in the two media (Figure 1G), the cell number was significantly lower in DMEM than in Plasmax. Altogether, these results illustrate that physiological levels of nutrients improve the metabolic fidelity of primary MSCs, prevent artefactual nutritional stress, enhance their stemness, and optimally support proliferation.

### 2.2 Bone marrow-relevant oxygen concentration transcriptionally rewires MSCs metabolism

**Figure 2:**
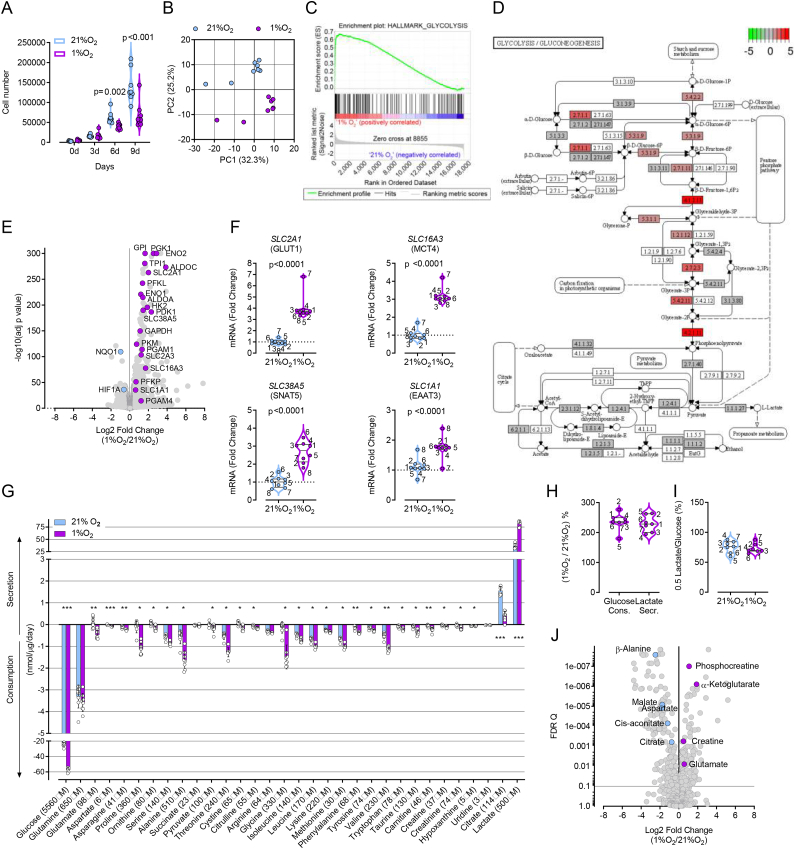
Bone marrow-relevant oxygen concentration transcriptionally rewires MSCs metabolism. (A) Cell numbers of MSCs incubated in Plasmax at 21% or 1% O_2_. Each data point represents one donor-derived cell line (n = 8 donors). p values refer to a two-tailed Student's t-test for paired samples. (B–I) MSCs were incubated for 48 h in Plasmax at 21% or 1% O_2_. (B) Principal component analysis obtained from the RNAseq analysis. Data points are means of values obtained with MSCs lines derived from 8 donors. (C) Enrichment plot for the “Glycolysis hallmark” gene set from GSEA. (D) Glycolysis/Gluconeogenesis KEGG pathway hsa00010 showing glycolytic genes identified by the RNAseq analysis (grey) as significantly upregulated (red) and downregulated (green) by 1% O_2_. Color scale indicates log_2_ fold change. (E) Volcano plot obtained from the RNAseq analysis. Labels are shown for a selection of metabolism-relevant genes significantly regulated by 1% O_2_. Data points are means of values obtained with MSCs lines derived from 8 donors. (F) Expression of four SLC encoding genes significantly regulated by 1% O_2_. Each data point represents one donor-derived cell line (n = 8 donors, labels indicate the donor identification number). Data points are expressed as fold change of the mean value obtained for the 8 cell lines cultured at 21% O_2_. p values refer to a two-tailed Student's t-test for paired samples. (G) Exchange rates (consumption and secretion) of metabolites between cells and media calculated as described in Materials and Methods. Data points are means ± SD of values obtained from 7 (for glucose and lactate) or 8 donor-derived MSCs lines (for all the other metabolites). ∗p < 0.05, ∗∗p < 0.01, ∗∗∗p < 0.001, p values refer to a two-tailed Student's t-test for paired samples corrected for multiple comparisons with the Holm-Sidak method. (H) Glucose consumption and lactate secretion at 1% O_2_ calculated as % of the value obtained at 21% O_2_. Each data point represents one donor-derived cell line (n = 7 donors). (I) Lactate secretion calculated as % of glucose consumption at 21% O_2_ and 1% O_2_. Lactate values were multiplied by 0.5 to reflect the glucose:lactate glycolytic molar ratio. Each data point represents one donor-derived cell line (n = 7 donors). (J) Volcano plot of intracellular metabolites with significantly different levels in cells incubated at 1% O_2_ compared to 21%O_2_. Blue and magenta circles represent metabolites, respectively, less and more abundant in cells cultured at 1%O_2_. Data points are means of values obtained with MSCs lines derived from 8 donors. q values refer to a false discovery rate approach corrected for multiple comparisons with a two-stage step-up method of Benjamini, Krieger and Yekutieli.

It has been shown that oxygen concentrations typical of the bone marrow (1–5%) enhance proliferation of MSCs when cultured in historic cell culture conditions [25]. Since, the effects of low oxygen concentration on MSC biology have not been investigated in physiological nutrient availabilities, we assessed the effect of hypoxia (1% O_2_) in a panel of donor-derived MSCs cultured in Plasmax. All cell lines decreased their proliferation rate in hypoxia, with an average doubling time of 52 and 72 h at 21% and 1% oxygen, respectively (Figure 2A). Next, we analyzed the effect of hypoxia on the transcriptome of all the MSCs by whole-transcriptome RNA sequencing. A principal component (PC) analysis of the results separated cells grown at different oxygen concentrations, showing that the variance attributable to oxygen availability exceeds that of inter-individual variability (Figure 2B). A gene set enrichment analysis (GSEA) [26] identified “hypoxia” and “glycolysis” (Figure 2C) as the gene sets with the highest enrichment scores (0.78 and 0.67 respectively), indicating an increased flux of glucose fermentation to lactate under hypoxic conditions.

Genes significantly regulated by hypoxia were mapped onto the “Glycolysis/Gluconeogenesis” KEGG pathway [27] visualizing the upregulated reactions in the pathway (Figure 2D). The volcano plot in Figure 2E highlights that hypoxia promotes gene transcription rather than repression, with 623 genes upregulated and 100 downregulated. Amongst the genes repressed by hypoxia we found *HIF1A*, consistent with transcriptional downregulation in prolonged hypoxia reported in other cell types [28]. Another gene whose expression was repressed by hypoxia is that encoding NAD(P)H quinone dehydrogenase 1, *NQO1*, a gene previously employed as reporter for the mitochondrial activity of MSCs [29]. Conversely, the expression of the GLUT1 glucose transporter encoding gene, *SLC2A1,* was increased 4-fold (Figure 2F), facilitating the uptake of glucose and its conversion to lactate, whose carrier *SLC16A3* (MCT4) was also significantly upregulated by hypoxia (Figure 2F). In addition, the mRNA level of the neutral amino acid transporter *SLC38A5* (SNAT5), as well as that of the high affinity glutamate transporter *SLC1A1* (EAAT3), was also significantly increased in hypoxia (Figure 2F). To assess the functional consequences of the transcriptional activation of metabolic genes in response to hypoxia, we quantified the exchange rates of nutrients and metabolites in 21% and 1% oxygen in the eight primary lines of MSCs cultured in Plasmax (Figure 2G). Consistent with the upregulation of glycolytic enzymes and glucose and lactate transporters, the rates of glucose consumption and lactate secretion were increased by more than two-fold at 1% oxygen (Figure 2G–H). However, the proportion of glucose converted to lactate (∼80%) remained unaffected in hypoxia (Figure 2I), demonstrating that the hypoxia-dependent transcriptional activation increases the flux of carbons through glycolysis without substantially increasing the relative fraction diverted towards alternative pathways. Differently from glucose and glutamate, whose consumption paralleled the increase in expression of their respective transporters (Figure 2F–G), the consumption of glutamine was uncoupled from *SLC38A5* expression and not increased in hypoxia (Figure 2F–G). Although less prominent than at 21% oxygen, the net efflux of citrate in the extracellular environment was maintained in hypoxic conditions, despite the impairment of the mitochondrial production of citrate. In fact, the intracellular level of citrate was decreased in hypoxia (Figure 2J), and this was consistent with the increased expression of *PDK1* that inhibits the pyruvate dehydrogenase (PDH)-dependent entry of pyruvate carbons into the TCA cycle (Figure 2E). Malate and aspartate, two metabolites linked to the mitochondrial oxidative activity, were also significantly decreased in hypoxia (Figure 2J). Conversely, α−ketoglutarate and glutamate levels were higher in hypoxia, showing that the pool of these two metabolites does not directly correlate with the amount of glucose carbons entering the TCA cycle and suggesting that the transamination of medium-derived glutamate partially compensates the deficit in TCA cycle activity.

### 2.3 Hypoxia diverts the flux of glucose carbons from the mitochondria towards the pentose phosphate pathway and lactate

**Figure 3:**
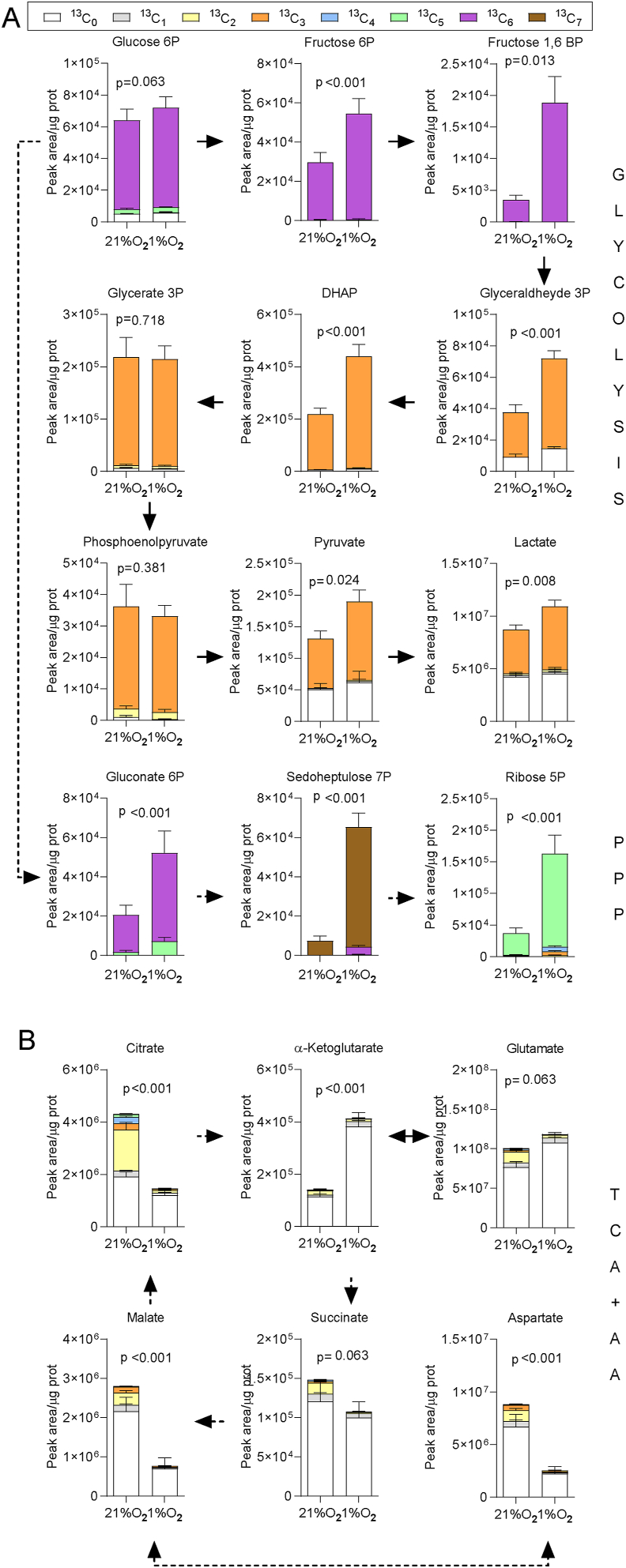
Hypoxia diverts the flux of glucose carbons from the mitochondria towards the pentose phosphate pathway and lactate. (A–B) MSCs were incubated for 48 h in Plasmax at 21% or 1% O_2_ in the presence of 5.56 mM ^13^C_6_ glucose. The relative isotopologue levels of intermediates of glycolysis and pentose phosphate pathway (A), and TCA cycle intermediates, glutamate and aspartate (B) are shown. Dashed arrows indicate conversions involving enzymatic reactions not shown. Bars are means ± SD of values obtained from 8 donor-derived MSCs lines. p values refer to a two-tailed Student's t-test for paired samples corrected for multiple comparisons with the Holm-Sidak method applied to the sum of all the indicated isotopologues. DHAP, Dihydroxyacetone phosphate; PPP, Pentose Phosphate Pathway; TCA, Tricarboxylic Acid; AA, Amino acids.

To investigate the metabolic fate of glucose in the eight MSCs lines cultured at 21% or 1% oxygen, where glucose consumption was increased (Figure 2G–H), we performed a stable isotope tracing experiment in Plasmax. The entire pool of glucose in the medium (5.56 mM) was replaced with ^13^C_6_ glucose. Consistently with the higher expression of the glucose transporters *SLC2A1* and *SLC2A3*, the fraction of ^13^C_6_ glucose-6- phosphate was higher at 1% than at 21% oxygen (Figure 3A). The total levels of the upper glycolytic intermediates, as well as their enrichment in heavy carbons from glucose, were markedly increased in hypoxia (Figure 3A). The increased flux in the upper glycolysis favored the branching of glucose carbons to the pentose phosphate pathway, as indicated by the marked increase in the levels of glucose-derived gluconate-6-phosphate, sedoheptulose-7-phosphate, and ribose-5-phosphate. The total levels and the heavy carbon enrichment of glycerate-3-phosphate and phosphoenolpyruvate were instead unchanged by hypoxia, suggesting that the activity of glyceraldehyde-3-phosphate dehydrogenase (GAPDH) could constitute a pinch point in the glycolytic flow. Nonetheless, the levels of the glucose-derived pyruvate and lactate were increased in hypoxia (Figure 3A), a result explained, at least in part, by the inactivation of the PDH mitochondrial complex. Indeed, hypoxia efficiently prevented the entry of glucose-derived carbon into the TCA cycle as indicated by the negligible levels of heavy citrate, α−ketoglutarate, malate, succinate and aspartate (Figure 3B). Consistently with the enrichment in glucose-derived carbons, the total levels of citrate, malate, succinate and aspartate were also decreased in hypoxia (Figure 3B). However, despite the lack of glucose carbon contribution to its pool, total α−ketoglutarate levels increased >3 fold in hypoxia. Consistently with the α−ketoglutarate levels, glutamate levels were also higher in hypoxia, suggesting that under this condition glutamate, either derived from glutamine or taken up from the medium, has an anaplerotic function.

### 2.4 Reductive carboxylation and glutamate anaplerosis sustain MSCs citrate synthesis and secretion in hypoxia

**Figure 4:**
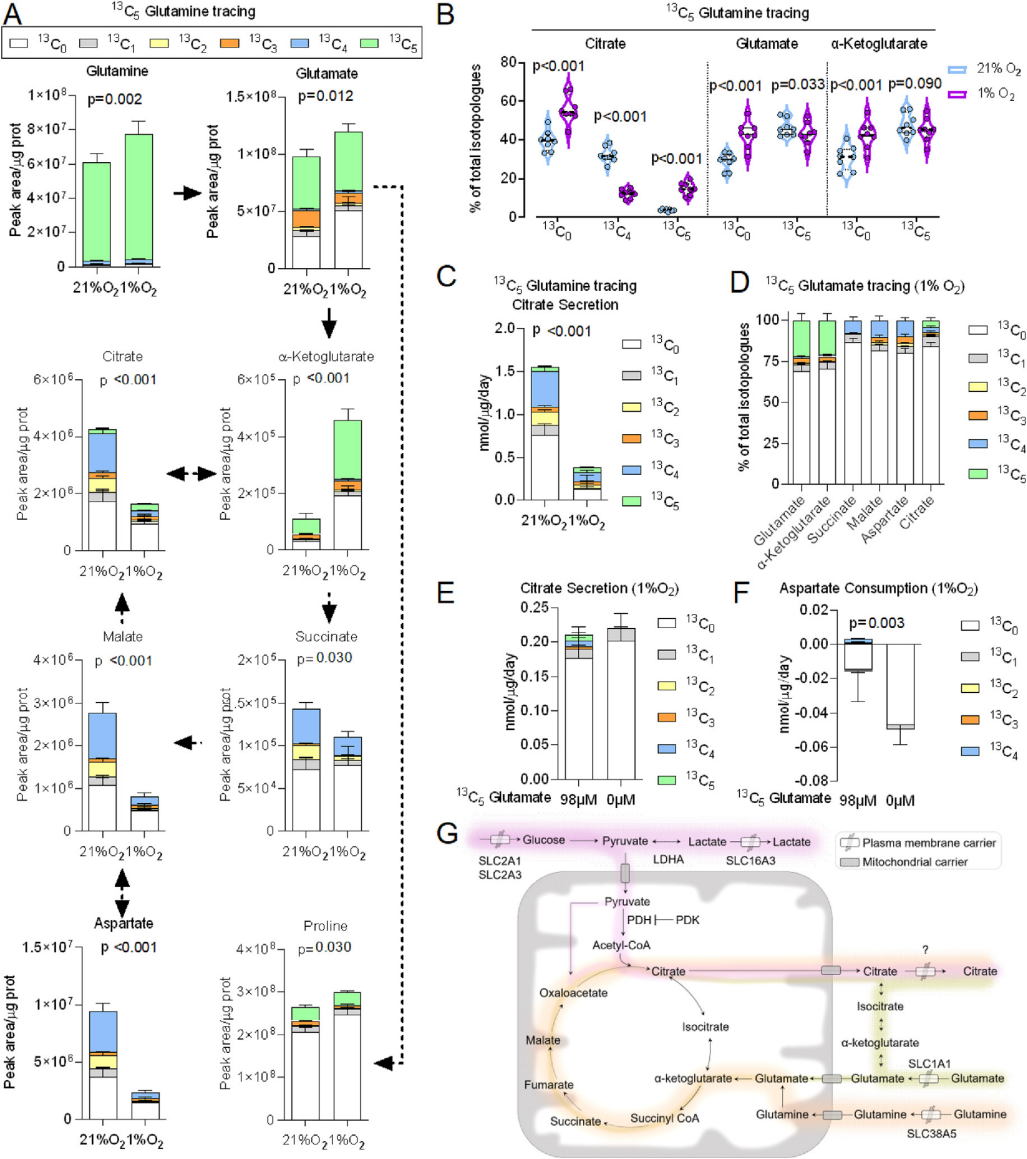
Reductive carboxylation and glutamate anaplerosis sustain MSCs citrate synthesis and secretion in hypoxia. (A–C) MSCs were incubated for 48 h in Plasmax at 21% or 1% O_2_ in the presence of 0.65 mM ^13^C_5_ glutamine. (A) The relative isotopologue levels of intermediates of glutamine metabolism and the TCA cycle are shown. Dashed arrows indicate conversions involving enzymatic reactions not shown. Bars are means ± SD of values obtained from 8 donor-derived MSCs lines. p values refer to a two-tailed Student's t-test for paired samples corrected for multiple comparisons with the Holm-Sidak method applied to the sum of all the indicated isotopologues. (B) Relative levels of the intracellular isotopologues of citrate, glutamate and α-ketoglutarate. Each data point represents one donor-derived cell line (n = 8 donors). p values refer to a two-tailed Student's t-test for paired samples corrected for multiple comparisons with the Holm-Sidak method. (C) The relative isotopologue levels of the citrate secreted in the medium. Bars are means ± SD of values obtained from 8 donor-derived MSCs lines. p value refers to a two-tailed Student's t-test for paired samples applied to the sum of all the indicated isotopologues. (D) MSCs were incubated for 48 h at 1% O_2_ in Plasmax where the entire pool of glutamate (0.098 mM) was replaced with ^13^C_5_ glutamate. The relative isotopologue levels of glutamate-derived metabolites are shown. Bars are means ± SD of values obtained from 4 donor-derived MSCs lines. (E–F) The relative citrate and aspartate isotopologue levels in spent media of cells incubated for 48 h at 1% O_2_ in Plasmax with no glutamate or with 0.098 mM ^13^C_5_ glutamate. Bars are means ± SD of values obtained from 4 donor-derived MSCs lines. p values refer to a two-tailed Student's t-test for paired samples applied to the sum of all the indicated isotopologues. (G) A simplified map of the main metabolic pathways engaged by MSCs cultured with physiological nutrients (Plasmax) and oxygen (1%) levels. Glucose, glutamine and glutamate dependent pathways are rendered with different colors.

To address the contribution of glutamine to mitochondrial metabolism in hypoxia, we traced physiological levels of ^13^C_5_ glutamine (0.65 mM) for 48 h. At this time point, the entire glutamine pool was replaced with the tracer and was significantly larger in MSCs cultured at 1% than at 21% oxygen (Figure 4A). Consistently with the tracing of heavy glucose, the pool of TCA cycle intermediates was smaller in hypoxia than at 21% oxygen. Unexpectedly, the shortage of glucose flux to mitochondrial oxidative metabolism caused by hypoxia was not compensated by glutamine's carbon contribution, as indicated by the smaller fraction of glutamine-derived carbons incorporated into citrate, malate, succinate, aspartate and proline (Figure 4A). However, the contribution of glutamine-derived carbons to citrate synthesis *via* the reductive carboxylation of α-ketoglutarate increased in hypoxia, as indicated by the intracellular and secreted fractions of ^13^C_5_ citrate (Figure 4B–C). Finally, the glutamine-to-glutamate conversion was largely unaffected by oxygen concentration, as indicated by the comparable levels of ^13^C_5_ glutamate found at 1% and 21% oxygen (Figure 4B). Conversely, the ^13^C_0_ fractions of glutamate and α-ketoglutarate were significantly larger in hypoxia, each accounting for 43% of the respective total pools (Figure 4B). Overall the results obtained by tracing ^13^C_6_ glucose and ^13^C_5_ glutamine suggested that in hypoxia the pool of TCA cycle intermediates could be repleted by glutamate transported from the extracellular environment. To test this hypothesis we incubated MSCs in Plasmax with 98 μM ^13^C_5_ glutamate for 48 h and measured the incorporation of heavy carbons in TCA cycle intermediates. One quarter of the intracellular pool of glutamate and its product α-ketoglutarate were ^13^C_5_ (Figure 4D), while 8% of succinate and 10% of malate and aspartate pools were ^13^C_4_. The distribution of heavy carbons from glutamate into citrate (∼2% ^13^C_3_, ∼3% ^13^C_4_, and ∼4% ^13^C_5_) indicates that the reductive carboxylation and oxidative decarboxylation of glutamate-derived alpha ketoglutarate contribute comparably to the synthesis of citrate. In addition we found that in hypoxia the contribution of extracellular glutamate to the secreted citrate was ∼12% of the total pool (^13^C_3-5_ citrate, Figure 4E), demonstrating that glutamate uptake contributes to citrate secretion. Finally, we tested whether glutamate withdrawal could impair citrate secretion, but no significant differences in the levels of secreted citrate were observed between cells incubated in glutamate-free or control Plasmax (Figure 4E). In glutamate-free Plasmax the consumption of aspartate was significantly increased (Figure 4F), possibly due to the absence of other high affinity substrates for the glutamate aspartate transporter *SLC1A1* (EAAT3).

All in all these results demonstrate that in hypoxia glutamate uptake from the extracellular environment feeds the intracellular pool of glutamate sustaining anaplerosis and citrate secretion (Figure 4G).

## 3 Discussion

Overall, this study demonstrates that primary MSCs can be expanded in physiological culture conditions that recapitulate the nutritional environment of the bone marrow better than DMEM and atmospheric oxygen concentrations. DMEM is the most widely employed medium in biomedical research and it is used in ∼60% of all the scientific papers that mention cell culture media [30]. MSCs cultures are no exception, and DMEM supplemented with protein factors has been proposed as the gold standard for the production of clinical-grade MSCs [31]. However, DMEM, as most of the historic blockbuster culture media, is not designed to recapitulate *in vitro* the physiological environment of human cells. Widely employed, yet nutritionally skewed, media directly affect the metabolism of cultured cells, and in turn signaling pathways, gene expression, and cellular functions [9,11,32]. By directly comparing the metabolism of primary MSCs cultured in DMEM and Plasmax, we showed that nutrients normally consumed by cells from the medium, such as alanine, proline, and uridine, are instead released by cells cultured in DMEM as a result of an artificially-imposed concentration gradient (Figure 1A). In addition, the lack of non-essential amino acids in DMEM causes nutritional stress and activates the GCN2-eIF2alpha-ATF4 signaling axis, boosting the synthesis of serine and asparagine (Figure 1A–C). A similar metabolic response has been observed in MSCs when treated with the antileukemic drug asparaginase [33], suggesting that culturing cells in DMEM recapitulates, at least in part, the metabolic effects triggered by the antileukemic enzyme. We also demonstrate that decreasing oxygen availability to physiological levels has profound effects on the metabolic wiring of MSCs, resulting in enhanced glycolysis (Figure 2G–H) and decreased mitochondrial oxidative metabolism (Figure 3B). Our results show that, differently from cancer cells [34], MSCs preferentially increase the consumption of neutral amino acids other than glutamine when exposed to low oxygen concentrations (Figure 2G). However, intracellular level of glutamine was significantly increased in MSCs cultured at 1% oxygen. This result is in line with the increased expression of *SLC38A5* (SNAT5) observed at 1% oxygen (Figure 2F). Glutamine is a preferential substrate of SNAT5 that couples its uptake with a stoichiometric proton extrusion. This transport mechanism could be advantageous at the intracellular acidic pH produced by the increased glucose fermentation observed at low oxygen concentration [35].

We found that glutamate was amongst the amino acids preferentially consumed in hypoxia by MSCs. This observation is coherent with the increased expression of the high affinity concentrative glutamate and aspartate transporter *SLC1A1* (EAAT3), whose activity in MSCs has not been reported before. Consistently, the intracellular total level of glutamate, α-ketoglutarate (Figure 2J), as well as their fractions not derived from glucose or glutamine (^13^C_0_, Figures 3B, 4A-B) increased in hypoxia, possibly favoring α-ketoglutarate-dependent histone hypo-methylation [36] and pluripotency [37,38].

Our results, in line with previous reports [39], show that MSCs are highly glycolytic (Figure 2G–I). Interestingly, at 21% oxygen citrate is the TCA cycle intermediate with the greates proportion of glucose-derived carbons (i.e. ^13^C_2_ Citrate in Figure 3B), but citrate synthesized from acetyl-CoA is preferentially diverted to the cytosol rather than being oxidized in the mitochondria (Figure 4G). This evident truncation of the TCA cycle, could be interpreted as an adaptation to sustain the here reported citrate secretion by MSCs. Furthermore, a comparative analysis of ^13^C_6_ glucose, ^13^C_5_ glutamine and ^13^C_5_ glutamate revealed that in hypoxia glutamine contributes most carbon atoms to citrate synthesis (^13^C_2-5_ citrate from glutamine ∼40%, Figure 4A) with glucose and glutamate contributing comparably (^13^C_2-5_ citrate from glucose ∼11% Figure 3B, and ^13^C_2-5_ citrate from glutamate ∼10% Figure 4D). However, rather than being utilized for fatty acids biosynthesis as shown for other cell types [40], cytosolic citrate is secreted by MSCs in the extracellular environment both in normoxia and hypoxia (Figure 4C,E). Remarkably, glutamate withdrawal did not significantly affected the amount of *de novo* synthesized citrate in the medium, suggesting that active citrate secretion is a dominant metabolic feature of MSCs that drives metabolic fluxes. In fact, we found that aspartate, the alternative substrate of *SLC1A1*, was significantly more consumed in the absence of glutamate than in control physiological conditions (Figure 4F).

Physiologically, high citrate is needed for bone matrix deposition [41], suggesting that MSCs contribute to bone renewal by providing the citrate in the extracellular environment. Moreover, it has been shown that citrate can be taken up and used as energy source by cancer cells [42,43] suggesting that MSC-derived extracellular citrate could sustain the metabolism of hematological cancer cells in the bone marrow niche as described for other metabolites [44].

In summary, we show that MSCs maintained in physiologically-relevant nutritional and hypoxic conditions take up glutamate from the extracellular environment through active, high affinity transport to sustain TCA cycle, as well as citrate synthesis and secretion. These previously unreported metabolic features can shape the metabolic bone marrow niche, representing potentially tractable targets in conditions such as osteoporosis, bone metastases and hematologic cancers.

## 4 Materials and Methods

### 4.1 MSC donors

Bone-marrow mesenchymal stromal cells (MSCs) were isolated from bone marrow of 9 male and 1 female donors aged 7.5 ± 4.2 years at the Pediatric Department of Fondazione MBBM/San Gerardo Hospital (Monza, Italy) as previously described [45]. Donors were enrolled in the AIEOP-BFM ALL 2009 protocol (EudraCT-2007-004270-43), and BCP-ALL/NICHE protocol, approved by “Comitato Etico Brianza” Ethical Committee. Informed consent to participate to the study was obtained for all subjects in accordance with the Declaration of Helsinki. Characteristics of donors are detailed in Table S1.

### 4.2 Cell culture

MSCs were grown for two passages from isolation in low-glucose Dulbecco's modified Eagle medium (DMEM), supplemented with 2 mM glutamine, 10% FBS and antibiotics (100 U/ml penicillin, 100 μg/ml streptomycin). Cells were screened for the expression of differentiation markers by flow cytometry, and were positive for CD73, CD90, CD105 and negative for CD45, CD14 and CD34, and were screened for their ability to differentiate into osteoblasts and adipocytes. MSCs at passage 2 were thawed in DMEM supplemented with 2 mM glutamine or thawed in Plasmax™ [9; 11] both supplemented with 10% FBS and antibiotics and maintained at 37 °C, pH 7.4 and 5% CO_2_. To evaluate the effects of different media on cell growth, MSCs were seeded at the density of 3,000 cells/cm^2^ in DMEM or Plasmax™, and counted every 72 h, using a Casy cell counter. To study the effects of hypoxia MSCs were cultured in Plasmax™ at 5% CO_2_ at 37 °C in water-saturated air at atmospheric oxygen (21% O_2_) or in a Whitley H35 Hypoxystation (Don Whitley Scientific) for hypoxic conditions (1% O_2_), 5% CO_2_ at 37 °C in water-saturated air.

### 4.3 RT-PCR

Cells were thawed and cultured as described in the “Cell Culture” section. For RT-PCR analysis MSCs were seeded at the density of 10,000 cells/cm^2^ in twelve-well plates in 0.8 ml DMEM (2 mM glutamine) or Plasmax™, both supplemented with 10% FBS and antibiotics. After 24 h, media were replaced with 3.2 ml of fresh medium, supplemented with 10% dialyzed FBS and antibiotics. After further 48 h, total RNA was isolated with GeneJET RNA Purification Kit (Thermo Fisher Scientific), and reverse transcribed with RevertAid RT Reverse Transcription Kit (Thermo Fisher Scientific). For real time qPCR, cDNA was amplified in a StepOne™ Real-Time PCR System (Applied Biosystems) employing a PowerUp™ SYBR™ Green Master Mix (Thermo Fisher Scientific) with 5 pmol of the primers indicated in the key resources table. The reaction consisted of 35 cycles including a denaturation step at 95 °C for 30 s, followed by separate annealing (30 s) and extension (30 s, 72 °C) steps. Fluorescence was monitored at the end of each extension step. At the end of the amplification cycles, a melting curve analysis was performed. Data were analysed according to the relative standard curve method. The mRNA expression of *SOX2, POU5F1, NANOG* and *RPL15,* was evaluated with the specific Taqman® gene expression assay (Thermo Fisher Scientific) indicated in the key resources table. Data were normalized to the expression of the housekeeping gene *RPL15*.

### 4.4 RNA sequencing

MSCs were seeded at 10,000 cells/cm^2^ in twelve-well plates in 0.8 ml of Plasmax™. After 24 h, medium was replaced with 3.2 ml of fresh Plasmax™, supplemented with 10% dialyzed FBS and antibiotics, and cells were incubated at 21% or 1% O_2_ as described in the “Cell culture” section. After 48 h, cells were harvested for RNA extraction and samples were processed using the QIAshredder/RNeasy Mini Kit (Qiagen). RNA samples were processed and sequenced as described previously [9]. Fastq files were generated from the sequencer output using Illumina's bcl2fastq and quality checks on the raw data were done using FastQC [46] and Fastq Screen [47]. The RNA-Seq paired-end reads were aligned to the GRCh38 [48] version of the human genome and annotation using Hisat2 [49]. Expression levels were determined and statistically analysed by a workflow combining HTSeq [50], the R environment [51] utilising packages from the Bioconductor data analysis suite [52] and differential gene expression analysis based on the negative binomial distribution using the DESeq2 package [53]. Further data analysis and visualisation used R and Bioconductor packages. Pathway and gene sets enrichment analysis were performed using the KEGG database [27] and GSEA [26].

### 4.5 Targeted metabolic analysis and metabolites’ exchange rates

Cells were thawed and cultured as described in the “Cell Culture” section. For the metabolic analysis cells were seeded at a density of 10,000 cells/cm^2^ in twelve-well plates with 0.8 ml of DMEM (2 mM glutamine) or Plasmax™. After 24 h (day 0), media were renewed with fresh medium, supplemented with 10% dialyzed FBS and antibiotics. The volume of medium/well was 0.8 ml for the determination of the exchange rates and 3.2 ml for the determination of intracellular metabolite levels. Cells were incubated at 21% or 1% O_2_, as described in the “Cell culture” section.

After a further 48 h (day 2), cells were washed three times with ice-cold PBS and the intracellular metabolites were extracted after 5 min of incubation at 4 °C in 200 μl of a methanol, acetonitrile, water solution (5:3:2). Cell extracts were centrifuged at 16,000*g*, 4 °C, for 10 min, and the content of metabolites in the supernatants were measured through LC-MS analysis performed as previously described [9]. Data were normalized to the protein content of each extracted well as quantified with the modified Lowry assay. For the determination of the exchange rates at day 2, the medium was harvested and diluted 1:50 in a solution of methanol, acetonitrile, water (5:3:2). Reference medium was also collected after an incubation of 48 h in parallel cell-free wells. Media were centrifuged at 16,000*g*, 4 °C, for 10 min, and the supernatants were collected for LC-MS analysis, performed as previously described [9]. Briefly, a Q Exactive Plus Orbitrap Mass Spectrometer (Thermo Fisher Scientific) was coupled with a Thermo Ultimate 3000 high performance liquid chromatography (HPLC) system. Five μl per sample were injected, and both positive and negative ions across a mass range of 75–1000 *m*/*z* were detected. Metabolites were separated with a mobile phase gradient of 15 min for a total run time of 23 min, and then quantified. Data were acquired through the software Xcalibur™ (Thermo Fisher Scientific). The peak areas of metabolites were determined by using the exact mass of the singly charged ions, and identity was confirmed by comparing experimental retention time (RT) to RTs predetermined by analysing an in-house mass spectrometry metabolite library. TraceFinder 4.0™ (Thermo Fisher Scientific) or the following versions were used for analysis. The protein content in each extracted well was quantified using a modified Lowry assay. Protein content of parallel wells seeded with the same number of cells were also determined at day 0. A five-point standard calibration curve (0.25x, 0.5x, 1x, 2x, 4x) was obtained by diluting or supplementing Plasmax components to glucose-free Earle's Balanced Salt Solution (EBSS) and used for the absolute quantification (μM or nmol) of metabolites in media. The exchange rate per day (positive values for secretion and negative values for consumption) for each metabolite (x) was calculated using the following equation:

$$x=\frac{\left(\text{nmol in spent medium }-\text{ nmol in cell free medium}\right)}{\left(\text{μg prot day }0+\text{μg prot day }2\right)/2}$$

### 4.6 Untargeted metabolomics

MSCs were seeded and harvested as described for the targeted metabolic analysis. Data analysis was performed using Compound Discoverer software (Thermo Scientific v3.2). Retention times were aligned across all data files (maximum shift, 2 min; mass tolerance, 5 ppm). Unknown compound detection (minimum peak intensity of 1 × 10^6^) and grouping of compounds were carried out across all samples (mass tolerance, 5 ppm; retention time (RT) tolerance, 0.7 min). Missing values were filled using the software's “Fill Gap” feature (mass tolerance, 5 ppm; signal/noise tolerance, 1.5). Compound identification was assigned by matching the mass and retention time of observed peaks to an in-house library generated using metabolite standards (mass tolerance, 5 ppm; RT tolerance, 0.5 min) or by matching fragmentation spectra to mzCloud (www.mzcloud.org, precursor and fragment mass tolerance, 10 ppm; match factor threshold, 60).

### 4.7 ^13^C_6_ glucose ^13^C_5_ glutamine and ^13^C_5_ glutamate tracing

MSCs were seeded and harvested as described for the targeted analysis. After 24 h, medium was substituted with Plasmax™, supplemented with 10% dialyzed FBS and antibiotics, containing U–^13^C_6_ Glucose (5.56 mM), U–^13^C_5_ glutamine (0.65 mM) or U–^13^C_5_ Glutamate (0.098 mM) obtained from Cambridge Isotopes Laboratories, MA, USA. For the determination of intracellular metabolite levels or exchange rates, 3.2 ml or 0.8 ml of medium were used, respectively. After 48 h of incubation at 21% or 1% O_2_, intracellular metabolites were extracted, and media samples were processed as described in the targeted metabolic analysis. For the U–^13^C_5_ Glutamate tracing experiments the 20 mM ammonium carbonate buffer used for the chromatography was supplemented with 5 μM medronic acid to improve the chromatographic resolution of citrate [54].

### 4.8 Immunoblot

Cells were seeded in Plasmax at 1 × 10^5^ cells/well in 6 wells plates. 24 h later, the medium was replaced with Plasmax or DMEM for further 48 h. Cells were then washed twice with ice-cold PBS and proteins were extracted using RIPA buffer (20–188, EMD Millipore Corp) containing proteases and phosphatases inhibitor cocktails (Complete, A32961, Thermo Fisher Scientific). Protein quantification was carried out using a bicinchoninic acid assay (A32961, PierceTM). Equal amounts of protein extracts (30 μg/lane) were loaded on 9.5% acrylamide gel for electrophoresis and blotted onto nitrocellulose membrane. PageRuler™ Prestained Protein Ladder (26,616, ThermoFisher scientific) was used as reference for molecular weight. The membrane was incubated overnight at 4 °C with the primary antibodies: anti-ATF4 (1:1000; ab184909, Abcam) and anti-β-actin (1:1000; ab8229, Abcam). Thereafter, the membrane was incubated for 1 h at room temperature with the secondary antibodies: anti-Rabbit-HRP (1:500; 7074, Cell Signaling Technology) or Anti-Goat-IRDye 800CW (1:15,000; 926–32214, Licor). The proteins were visualized with an Odyssey infrared scanner (Licor) or Chemidoc MP imager (Biorad) using Clarity Western ECL Substrate (1,705,061, Biorad). Band intensities were quantified with Image Studio Lite and Chemidoc MP imager (Biorad).

### 4.9 Statistical analysis

Data were expressed as means ± SD as indicated in the figure legends. Data points representing MSCs from individual donors are indicated in the figure legends and shown when not impairing figure clarity. For the untargeted metabolomics, a false discovery rate approach corrected for multiple comparisons with a two-stage step-up method of Benjamini, Krieger and Yekutieli (Graph Pad Prism 9.2.0) was used for volcano plots. The statistical analysis for the RNA sequencing data is described in the specific section above. For all the other statistical analysis, GraphPad Prism 9.2.0 or newer versions were used. Statistical tests were employed as specified in the figure legends. Statistical significance is reported in figures with exact p values or defined in the figure legends.

### 4.10 Data and reagents availability

RNA-seq data have been deposited in the ArrayExpress database at EMBL-EBI (www.ebi.ac.uk/arrayexpress) under accession number E-MTAB-11421. Reagents and software are listed in Table S2.

## Author contributions

Conceptualization: G.T., O.B., S.T.; Methodology: D.S., E.S., R.S., A.H.; Data Curation: R.S., A.H.; Resources: ED, G. D’A.; Investigation: G.T., R.D., V.H.V., M.C.; Visualization: G.T., ST.; Funding acquisition: G.T., G. D’A., O.B., ST.; Supervision: O.B., ST.; Writing – original draft: G.T., S.T.; Writing – review & editing: G.T., M.C., O.B., S.T.
