# Supplementary material for: Mesenchymal stromal cells cultured in physiological conditions sustain citrate secretion with glutamate anaplerosis
Source: Mol Metab. 2022 Jun 22;63:101532. doi: 10.1016/j.molmet.2022.101532 (PMC9254159; doi:10.1016/j.molmet.2022.101532)
Supplement: Multimedia component 1 [file mmc1.pdf]

Mesenchymal stromal cells cultured in physiological conditions sustain citrate secretion with glutamate anaplerosis

Giuseppe Taurino[1,4], Ruhi Deshmukh[2], Victor H. Villar[2], Martina Chiu[1], Robin Shaw[2], Ann Hedley[2], Engy Shokry[2], David Sumpton[2], Erica Dander[3], Giovanna D'Amico[3], Ovidio Bussolati[1,4*], Saverio Tardito[2,5*]

[1]Laboratory of General Pathology, Dept. of Medicine and Surgery, University of Parma, 43125, Parma, Italy.

[2]Cancer Research UK Beatson Institute, Garscube Estate, Switchback Road, Glasgow, G61 1BD, UK

[3]Centro Ricerca Tettamanti, Pediatric Dept., University of Milano-Bicocca, Fondazione MBBM, Monza, 20900, Italy.

[4]MRH - Microbiome Research Hub, Parco Area delle Scienze 11/A, University of Parma, 43124 Parma, Italy.

[5]Institute of Cancer Sciences, University of Glasgow, Glasgow, G61 1QH, UK.

*Correspondence: ovidio.bussolati@unipr.it (OB), s.tardito@beatson.gla.ac.uk (ST)

Supplementary Material

Table S1 Details of the MSCs donors.

Table S2 Reagents and software table.

Table S1 Details of the MSCs donors.

Donor number	Age (years)	Sex
1	3	Male
2	10	Male
3	5	Male
4	11	Male
5	5	Male
6	5	Male
7	17	Male
8	6	Female
9	10	Male
10	3	Male

Table S2 Reagents and software table.

REAGENT	SOURCE	IDENTIFIER
**Chemicals**		
DMEM low-glucose, pyruvate, no glutamine	Euroclone<br>Sigma	Cat#ECM0749L<br>Cat#D5546
Plasmax™	Ximbio	Cat#156371
Fetal Bovine Serum (FBS)	Gibco	Cat#10270-106
L-Glutamine 200mM	Euroclone	Cat#ECB30000
L-Glutamine (U-^{13}C5, 99%)	Cambridge Isotopes Laboratories	Cat#CLM-1822-H-PK
L-Glutamate (U-^{13}C5, 98%)	Cambridge Isotopes Laboratories	Cat#CLM-1800
D-Glucose (U-^{13}C6, 99%)	Cambridge Isotopes Laboratories	Cat#CLM-1396
**Antibodies**		
Anti-ATF4	Abcam	Cat#ab184909
Anti-β-actin	Abcam	Cat#ab8229
Anti-Rabbit-HRP (	Cell Signaling	Cat#7074
Anti-Goat-IRDye 800CW	Licor	Cat#926-32214
**Experimental Models: Organisms/Strains**		
Human mesenchymal stromal cells from Donors (see Table S1)	Pediatric Department of Fondazione MBBM/San Gerardo Hospital (Monza, Italy)	N/A

**Oligonucleotides**		
Human *PSAT1* primers:	Sigma-Aldrich	For 5' CGGTCCTGGAATACAAGGTG 3'<br>Rev 5' AACCAAGCCCATGACGTAGA 3'
Human *DDIT3* primers:	Sigma-Aldrich	For 5' CTTCTCTGGCTTGGCTGACT 3'<br>Rev 5' TCCCTTGGTCTTCCTCCTCT 3'
Human *RPL-15* primers:	Sigma-Aldrich	For 5' GCAGCCATCAGGTAAGCCAAG 3'<br>Rev 5' AGCGGACCCTCAGAAGAAAGC 3'
Human *SOX-2*	Thermo Fisher Scientific	hs01053049_s1
Human *POU5F1*	Thermo Fisher Scientific	hs03005111_g1
Human *NANOG*	Thermo Fisher Scientific	hs02387400_g1
Human *RPL-15*	Thermo Fisher Scientific	hs03855120_g1
**Deposited Data**		
RNA-seq data	EMBL-EBI https://www.ebi.ac.uk/arrayexpress/experiments/E-MTAB-11421	E-MTAB-11421
**Software and Algorithms**		
GraphPad Prism 9.2.0™	GraphPad Software Inc	https://www.graphpad.com/scientific-software/prism/
TraceFinder 4.0™	Thermo Fisher Scientific	https://www.thermofisher.com/it/en/home/industrial/mass-spectrometry/liquid-chromatography-mass-spectrometry-lc-ms/lc-ms-software/lc-ms-data-acquisition-software/tracefinder-software.html
Xcalibur™ 4.3	Thermo Fisher Scientific	https://www.thermofisher.com/order/catalog/product/OPTON-30965
Compound Discoverer™ 3.2	Thermo Fisher Scientific	https://www.thermofisher.com/it/en/home/industrial/mass-spectrometry/liquid-chromatography-mass-spectrometry-lc-ms/lc-ms-software/multi-omics-data-analysis/compound-discoverer-software.html
Bcl2fastq 2.20.0.422	Illumina	RRID:SCR_015058

FastQC 0.11.7	Babraham Bioinformatics	RRID:SCR_014583
Fastq Screen 0.14.0	Babraham Bioinformatics	RRID:SCR_000141
HISAT 2.2.1		RRID:SCR_015530
HTSeq 0.11.2		RRID:SCR_005514
DESeq2 1.30.0	Bioconductor	RRID:SCR_015687
R environment 4.0.2	R Core Team, 2018.	https://www.R-project.org
GRCh38.95		https://genome.ucsc.edu/index.html
KEGG		https://www.genome.jp/kegg/pathway.html
GSEA		https://www.gsea-msigdb.org/gsea/index.jsp
